# Efficacy and safety of artemisinin-based combination therapy and chloroquine with concomitant primaquine to treat *Plasmodium vivax* malaria in Brazil: an open label randomized clinical trial

**DOI:** 10.1186/s12936-018-2192-x

**Published:** 2018-01-24

**Authors:** André Daher, Dhelio Pereira, Marcus V. G. Lacerda, Márcia A. A. Alexandre, Cristiana T. Nascimento, Júlio Castro Alves de Lima e Silva, Mauro Tada, Rosilene Ruffato, Ivan Maia, Tereza Cristina dos Santos, Paola Marchesini, Ana Carolina Santelli, David G. Lalloo

**Affiliations:** 1 Institute of Drug Technology (Farmanguinhos), Oswaldo Cruz Foundation (FIOCRUZ), Rio de Janeiro, Brazil; 2 Vice-presidency of Research and Reference Laboratories, Oswaldo Cruz Foundation (FIOCRUZ), Rio de Janeiro, Brazil; 3 Liverpool School of Tropical Medicine, Liverpool, UK; 4 Tropical Medicine Research Center of Rondonia (CEPEM), Porto Velho, Brazil; 5 Federal University of Rondonia (UNIR), Porto Velho, Brazil; 6 Research Institute Leônidas & Maria Deane, FIOCRUZ, Manaus, Brazil; 7 Tropical Medicine Foundation Dr Heitor Vieira Dourado, Manaus, Brazil; 8 National Institute of Infectious Disease, Oswaldo Cruz Foundation (FIOCRUZ), Rio de Janeiro, Brazil; 9 National Malaria Control Programme, Ministry of Health, Brasília, Brazil

**Keywords:** Malaria, *Plasmodium vivax*, Antimalarial treatment, Chloroquine, Mefloquine, Artesunate, Lumefantrine, Artemether, Primaquine, Artemisinin-based combination therapy, ACT, Clinical trial

## Abstract

**Background:**

There is general international agreement that the importance of vivax malaria has been neglected, and there is a need for new treatment approaches in an effort to progress towards control and elimination in Latin America. This open label randomized clinical trial evaluated the efficacy and safety of three treatment regimens using either one of two fixed dose artemisinin-based combinations or chloroquine in combination with a short course of primaquine (7–9 days: total dose 3–4.2 mg/kg) in Brazil. The primary objective was establishing whether cure rates above 90% could be achieved in each arm.

**Results:**

A total of 264 patients were followed up to day 63. The cure rate of all three treatment arms was greater than 90% at 28 and 42 days. Cure rates were below 90% in all three treatment groups at day 63, although the 95% confidence interval included 90% for all three treatments. Most of the adverse events were mild in all treatment arms. Only one of the three serious adverse events was related to the treatment and significant drops in haemoglobin were rare.

**Conclusion:**

This study demonstrated the efficacy and safety of all three regimens that were tested with 42-day cure rates that meet World Health Organization criteria. The efficacy and safety of artemisinin-based combination therapy regimens in this population offers the opportunity to treat all species of malaria with the same regimen, simplifying protocols for malaria control programmes and potentially contributing to elimination of both vivax and falciparum malaria.

*Trial registration* RBR-79s56s

**Electronic supplementary material:**

The online version of this article (10.1186/s12936-018-2192-x) contains supplementary material, which is available to authorized users.

## Background

Malaria control and elimination activities have mainly focused upon *Plasmodium falciparum* as the leading cause of malaria mortality and morbidity worldwide. Nevertheless, *Plasmodium vivax* remains a major public health problem [1]. In 2010, 2.48 billion people worldwide were living in areas at risk for vivax infection. Brazil is the largest endemic area in the Americas; where stable transmission and a dispersed population [2, 3] pose major challenges for malaria control [4, 5]. In 2014, 143,552 malaria cases were reported [6].

Over the last 30 years, the proportion of malaria cases due to vivax in Brazil has increased from 50%, in 1988 to 84% in 2014. *Plasmodium vivax* now causes 65% of hospitalizations due to malaria in the Brazilian Amazon region, and 13 out of 38 malaria deaths in Brazil in 2014 were due to *P. vivax* [6]. Control and management of this disease is therefore important, particularly in light of the increasing recognition that *vivax* infection is not as benign as first thought and that vivax can both cause severe disease [7–9], and have negative impacts on prosperity, longevity, school performance, pregnancy and the economy [10–12]. Optimizing the treatment of vivax malaria is critical to improve vivax control.

In Brazil, the current treatment recommended to treat vivax is chloroquine with concomitant use of 7 days of primaquine. The primaquine total dose ranges from 3 to 4.2 mg/kg [13]. Recent trials have not demonstrated chloroquine resistant vivax in Brazil [14–16], although there are still concerns about chloroquine resistance emergence [17]. The availability of other options, particularly ones that may be useful for the treatment of both vivax and falciparum malaria, would help to avoid selective pressure over a single therapeutic regimen. This study was designed to evaluate new approaches for the acute treatment of vivax in preparation for plans for both vivax elimination and updated treatment guidelines.

This randomized clinical trial evaluated the efficacy and safety of three vivax treatment regimens using either an ACT or chloroquine with concomitant use of primaquine in Brazil. The primary objective was to establish whether cure rates above 90% could be achieved in each arm [18].

## Methods

### Study population

Patients with uncomplicated vivax malaria were included in the study, after giving informed consent, if they met the following inclusion criteria: age between 18 and 70 years; weight between 50 and 90 kg, *P. vivax* mono-infection confirmed by microscopy, asexual parasite count > 250/μL, axillary temperature ≥ 37.5 °C or a history of fever during the past 48 h, and haemoglobin > 7.0 g/dL. Exclusion criteria were: malaria treatment in the previous 63 days; signs of severe malaria; concurrent other febrile conditions or chronic disease (such as severe cardiac, hepatic or renal disorders or HIV); the use of any medication known to interfere with anti-malarial pharmacokinetics; previous history of intolerance to any study drug; known glucose-6-phosphate deficiency; pregnancy confirmed by urinary human chorionic gonadotropin (hCG) testing; and breastfeeding.

### Study design and drug administration procedures

This prospective, randomized, open label, three-arm efficacy study of uncomplicated vivax malaria was conducted according to World Health Organization (WHO) methods for surveillance of anti-malarial drug efficacy [18] at two centres in the Amazon Region of Brazil: the Tropical Medicine Research Centre (CEPEM) in Rondônia and Tropical Medicine Foundation Dr Heitor Vieira Dourado (FMT-HVD) in Manaus.

A 90% cure rate or greater is considered by the WHO to be sufficient evidence of efficacy to support the choice of a specific regimen by National Malaria Control Programmes in their treatment guidelines [18]. The sample size was calculated with an expected failure rate of 5%. 88 patients were included in each study arm to achieve a precision of 5% and allowing for 20% loss to follow-up, leading to a total of 264 patients.

A randomization list using blocks of six and allocation rate 1:1:1 was generated by software (Etcetera, version 2.72). Sequentially numbered (0–176 CEPEM and 177–264 FMT) opaque sealed envelopes were provided to the local clinical coordinators and used to randomize patients. Differences in dosing schedules and the difficulty of dummy blinding meant that neither patients nor healthcare workers were blinded, but microscopists were not aware of treatment allocation. The statistician was blind to the treatment allocation until the database had been locked.

Eligible patients were allocated to one of the following three treatment groups: (a) chloroquine (CQ); (b) fixed dose combination of artesunate and mefloquine (ASMQ) and; (c) fixed dose combination of artemether and lumefantrine (AL). All three arms received the same primaquine regimen.

Group A received chloroquine (Farmanguinhos—Fiocruz, Batch Numbers 12080940 and 14060467) 600 mg on day 1, and 450 mg on days 2 and 3. This is the Brazilian Ministry of Health current recommendation for uncomplicated malaria vivax treatment [13].

Group B received two tablets daily for 3 days of a fixed dose combination of 100 mg + 200 mg artesunate and mefloquine (ASMQ) tablets (Farmanguinhos—Fiocruz Batch Numbers 11100680 and 13040348) in a total of six tablets.

Group C received four tablets twice a day for 3 days of a fixed dose combination of 20 mg + 120 mg artemether and lumefantrine (AL) tablets (Coartem^®^—Novartis, Batch Numbers F2618 and K30711) in a total of 24 AL tablets.

All groups also received two tablets of 15 mg primaquine (Pq) (Farmanguinhos—Fiocruz, Batch numbers 12010038 and 13030282) for 7, 8 or 9 days (according to three weight ranges; ≥ 50–69; 70–79; 80–90 kg, respectively), as recommended by National Malaria Control Programme treatment guidelines [13]. Patients received a total primaquine dose between 3.0 and 4.2 mg/kg.

The first 3 days’ treatment were supervised for Group A and B. For Group C, only the first daily AL dose was directly observed. Patients in the AL arm were asked about their adherence to the previous second daily dose after the morning supervised dose. For all groups, the first dose was administered after diagnosis, and all subsequent supervised doses were taken between 8 and 10 a.m. If the patient vomited within 30 min after a dose, the same dose was administered again. At D7 follow-up visit, patients were inquired about their treatment adherence to unsupervised primaquine on days 4 through 7. The use of other treatments was recorded at every visit.

Glucose-6-phosphate dehydrogenase (G6PD) deficiency screening was not performed: this is not routinely performed in Brazil and it is not required by the national treatment guidelines. At enrolment, patients were asked about adverse events during previous primaquine use.

### Ethics statement

The clinical study protocol and informed consent form were reviewed and approved by the Ethics Committee at CEPEM (No. 31/11 CEP/CEPEM 0018.0.046.000-11 CAAE—SISNEP and Plataforma Brasil No. 74869 CEP/CEPEM No. 05462612.7.0000.0011 CAAE). In April 2014, an amendment (No. 644.709 CEP/CEPEM) approved the inclusion of the second study site FMT-HVD, in Manaus. The Brazilian National Council on Ethics in Research (CONEP), Ministry of Health, accredits the CEPEM Ethics Committee. The study is registered at the Brazilian Register of Clinical Trials (RBR-79s56 s U1111-1132-8050), a primary repository of WHO. The clinical study was conducted in accordance with the Helsinki Declaration (Edinburgh, 2000), Good Clinical Practice [19, 20] and the Brazilian National Health Council (CNS) resolution 466/2011. During the study, monitoring visits were conducted to ensure GCP adherence. Written informed consent was obtained for every subject prior to enrolment. If the study subject was illiterate, an impartial third party witnessed the informed consent process. All subjects were informed of the nature and possible associated risks of the trial and that they were free to withdraw their consent to participate at any time. The investigators and study staff ensured confidentiality of all records.

### Efficacy and safety evaluations

Patients were assessed on the day of enrolment and on days 1, 2, 3, 7, 14, 21, 28, 42 and 63 days after study inclusion. The scheduled study procedures comprised a full history, physical examination, and urinalysis at enrolment and assessment for clinical signs and adverse events (AE) at every follow-up visit. Blood samples were collected for parasite counts at 0, 3, 7, 14, 21, 28, 42 and 63 days, and haemoglobin was measured at 0, 14, 28, 42 and 63 days. An additional blood smear was also collected whenever treatment failed. Data was double entered using an electronic clinical record form (OpenClinica Community, version 3.1.3.1). Analysis was performed using R (version 3.2.5).

The primary efficacy endpoint was the proportion of the population with an adequate clinical and parasitological response (ACPR) at 63 days. Early treatment failure or late treatment failure were classified in line with standard WHO methodology [18]. The primary analysis was per protocol (PP); an intention-to-treat (ITT) analysis was also performed. The PP population excluded any participant with a protocol violation. Patients who missed the 28 or 42-day visit, but who had an ACPR at the subsequent follow visit was considered as a success at the previous visit. In the ITT population, protocol violations or losses to follow-up were considered as parasitological and clinical failure.

The secondary efficacy endpoints included the success rate at day 3 (72 h after first drug administration) as well as gametocyte clearance, fever clearance, and the cumulative success rate at days 28, 42 and 63.

The cumulative success rate by day 63, i.e. the probability of remaining parasite-free at day 63, was calculated using a Kaplan–Meier survival curve. Categorical variables were summarized using frequencies and percentages, while for quantitative variables, means, standard deviations (SD) and maximum–minimum values were used. Parasite counts were presented using geometric means.

The study was not designed to compare outcomes between treatment arms. However, exploratory analyses were conducted to explore the impact of the treatment arm on the safety and efficacy outcomes. The proportions of categorical variables were compared using Pearson’s Chi squared test with Yates’ continuity correction at significance level of 5%. Nonparametric tests of Wilcoxon and Kruskal–Wallis were used for continuous variables. Additionally, generalized linear models (binominal and Poisson distribution, respectively) were used to estimate the effect of predictors (baseline characteristics and the use of other medications) on treatment success at day 63 and the numbers of AEs that were possibly, probably or highly probably related to the treatment.

A safety analysis was conducted in the ITT population describing frequency, causality, and severity of AEs in each treatment arm. AEs reports were subdivided into: serious AEs, AEs leading to treatment suspension, and AEs which were described as possibly, probably or highly probably related. The mean haemoglobin results at baseline and follow-up are also presented.

Patients were encouraged to seek unscheduled assessments if any AE was suspected. All clinical or laboratory abnormalities were categorized as Grade I to IV according to the Common Terminology Criteria for Adverse Events (CTCAE) of the National Cancer Institute [21]. Any suspected serious AE (standard definitions) was reported to the sponsor and the Ethical Review Committee. Recognized drug-related events were recorded as an AE, even if it could be related to malaria.

Parasitological densities were estimated using Giemsa-stained blood slides at a magnification of 1000× using WHO recommended methods [18]. Two trained microscopists read slides independently. The final density was calculated as the mean of the two readings. A third microscopist examined slides if the two readers disagreed over whether there were parasites present, the species, or the parasite density (more than 50% difference). In such cases, the final density was considered as the average of the two closest counts. A slide was considered as negative only after examining 1000 leucocytes in microscopic fields. Gametocyte presence was also recorded.

## Results

### Baseline characteristics of the study population

**Fig. 1:**
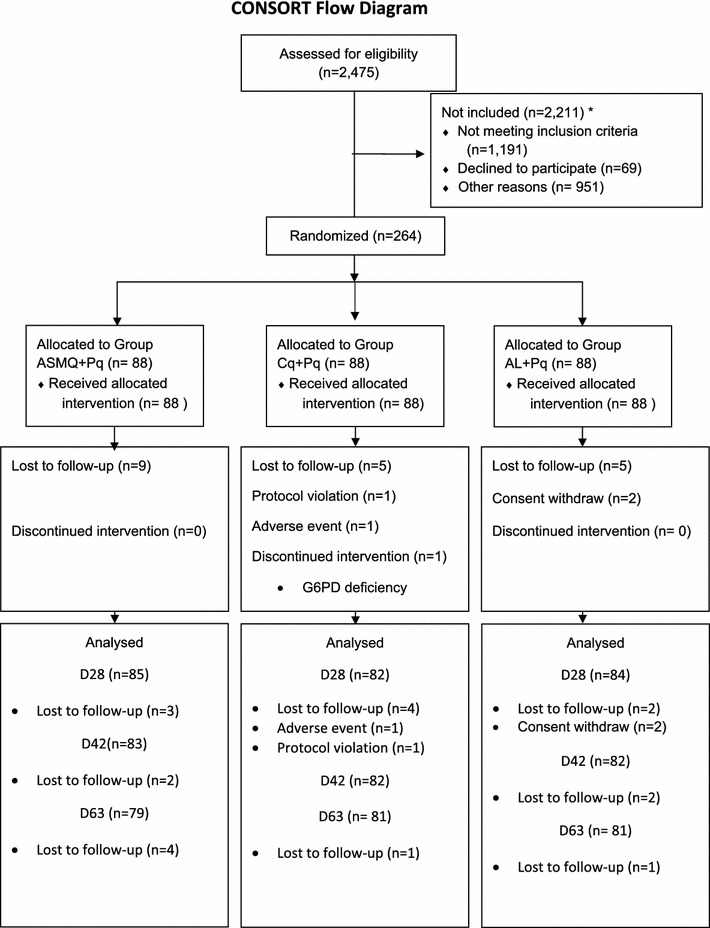
CONSORT flow diagram

A total of 2475 malaria thick smear positive patients were screened for inclusion in the two trial sites (CEPEM and FMT-HVD) from August 2012 to February 2015. 264 (10.7%) were randomized (1:1:1) to one of three different treatment arms: CQ + Pq; ASMQ + Pq and AL + Pq. The same number of patients was allocated to each arm. The main reasons for not including patients were unavailability for follow-up (19.9%), parasitaemia lower than 250/μL (15.5%) and malaria treatment within the past 63 days (15.2%). Figure 1 shows the CONSORT flow diagram, see Additional file 1 for reasons not to be included. Twenty-three patients did not complete the study; there was no difference in the proportions that discontinued between the arms. Only one protocol deviation occurred: the inclusion of a patient with a mixed infection, confirmed by PCR. The baseline characteristics of the patients were similar among the treatment groups (Table 1), and the ITT and PP populations.

**Table 1:** Baseline characteristics

Baseline characteristics	Treatment group		
	ASMQ + Pq	CQ + Pq	AL + Pq
N (%)	88 (33.3%)	88 (33.3%)	88 (33.3%)
Study site			
CEPEM	66 (75.0%)	66 (75.0%)	66 (75.0%)
FMT-HVD	22 (25.0%)	22 (25.0%)	22 (25.0%)
Gender			
Female	32 (36.4%)	22 (25.0%)	24 (27.3%)
Male	56 (63.6%)	66 (75.0%)	64 (72.7%)
Age (years)			
> 59	3 (3.4%)	4 (4.5%)	3 (3.4%)
18–39	45 (51.1%)	34 (38.6%)	49 (55.7%)
39–59	40 (45.4%)	50 (56.8%)	36 (40.9%)
Weight (kg)			
> 80	25 (28.4%)	22 (25.0%)	27 (30.7%)
50–65	27 (30.7%)	21 (23.9%)	23 (26.1%)
65–80	36 (40.9%)	45 (51.1%)	38 (43.2%)
Fever			
< 37.5	48 (54.5%)	54 (61.4%)	53 (60.2%)
> 37.5	40 (45.4%)	34 (38.6%)	35 (39.8%)
Weight (kg)	71.59 (10.99) [51–90]	72.7 (10.34) [53–90]	72.53 (10.59) [50–90]
Temperature	37.45 (1.38) [34.7–40.5]	37.31 (1.21) [35.0–39.9]	37.38 (1.22) [35.2–40.6]
Parasitaemia	2145 (2516.4) [258–13,340]	2155 (2908.5) [285–17,680]	2444 (3974.6) [270–20,080]
Age (years)	38.75 (10.9) [19.5–65.8]	41.88 (10.5) [19.7–64.9]	37.24 (11.8) [18.4–64.3]

Absolute number and percentage (%) were used to present categorical variables. Quantitative data presented using means, standard deviations (SD), and ranges [min–max]. Parasitaemia presented using geometric mean

There were 495 reports of concomitant medication use in the study. Analgesic and antipyretic were most frequently used (287 reports); there was no difference in use between the three arms. Use of anti-ulcer and antispasmodic drugs were more common in the AL arm; accounting for 40.5% (15/27) and 69.6% (16/23) of the patients that used these classes, respectively.

A table presenting the medication used (grouped by therapeutic class) in each study arm, and a figure illustrating the most commonly used medications used per study visit and treatment group are provided (see Additional file 2).

Treatment adherence was 100% for ASMQ and CQ, as the dose during the initial 3 days was supervised. Nineteen patients reported AL non-adherence to the second daily dose: 16 patients reported one missing dose, 2 patients reported two missing doses, and one patient missed doses on three occasions. However, only one patient with incomplete AL adherence presented a treatment failure at day 63. Patients were asked about Pq adherence at D7 visit. Only five patients reported incomplete treatment; two in each ASMQ + Pq and CQ + Pq arms and one in the AL + Pq arm. None of them presented treatment failure.

### Efficacy evaluation

The primary objective was to demonstrate cure rate above 90% in each study arm. The cure rate was defined as the proportion of the population with an adequate clinical and parasitological response (ACPR) at day 63. Cure rates were below 90% in all three treatment groups at day 63 in the PP population, although the 95% CI did include 90% for all three drugs: 85% (95% CI [77–93%]) of the ASMQ group, 88% (95% CI [81–95%]) of the CQ group, and 84% (95% CI [76–92%]) of the AL group achieved an ACPR at day 63. The cure rate of all the three treatment arms was greater than 90% at 28 and 42 days in the PP population (Table 2).

**Table 2:** Proportion of treatment success per treatment arm in PP population at day 28, 42 and 63

Visit day	Study treatment					
	ASMQ + Pq		CQ + Pq		AL + Pq	
	% (n)	95% CI	% (n)	95% CI	% (n)	95% CI
D28	100 (85)	–	100 (82)	–	96 (84)	92–100
D42	98 (83)	95–100	93 (82)	87–99	94 (82)	89–99
D63	85 (79)	77–93	88 (81)	81–95	84 (81)	76–92

**Fig. 2:**
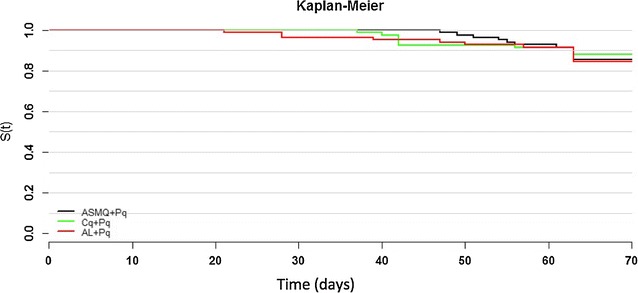
Kaplan–Meier survival analysis of the three treatment arms

Cure rates in the ITT analysis were slightly lower (see Additional file 3) predominantly reflecting the very conservative approach to missing data. Secondary efficacy endpoints included the success rate at day 3 (72 h after first drug administration) and the cumulative success rate at days 28, 42 and 63, calculated using a Kaplan–Meier survival curve. These results are summarized in Fig. 2.

Parasitological success rates at day 3 were 100% for all arms in both PP and ITT analyses, apart from the AL + Pq arm where two dropouts (one informed consent withdraw, and one lost to follow-up) meant that the ITT success rate at day 3 was 98% (95% CI [98–100]).

**Fig. 3:**
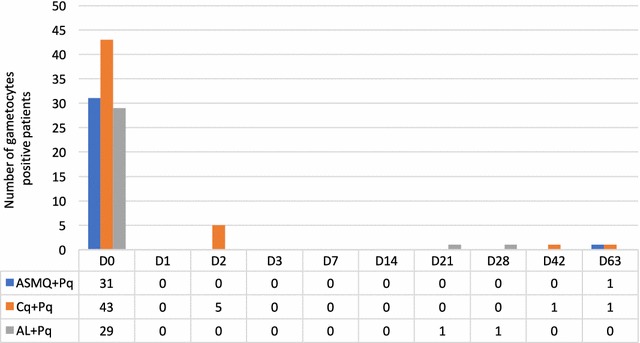
Clearance of gametocytes

Fever clearance is an important surrogate for cure in malaria. Only one patient in the CQ + Pq arm and two in the AL + Pq arm had an axillary temperature higher than 37.5 °C at day 3. Four fever episodes were reported after day 7. One patient had fever at day 63 and was considered as a treatment failure. The other three patients completed 63 days follow up without evidence of parasites on microscopy. All patients cleared gametocytes by day 3. The five patients that had gametocytes present on day 2 had all been treated with CQ + Pq (Fig. 3).

### Safety results

Three serious adverse events (SAE) were reported. One patient in the CQ arm had treatment suspended because of haemolytic anaemia on day 3. Qualitative calorimetric test confirmed glucose-6-phosphate dehydrogenase (G6PD) deficiency. Another in the AL arm had a rise in alanine aminotransferase (ALT) detected on day 12 after treatment. The third SAE reflected elective surgery and was not related to the trial. All patients recovered completely.

The safety analysis was conducted in the ITT population. A total of 1593 adverse events were reported. 1379 of them (86.5%) were classified as grade I and 208 events (13.1%) were classified as grade II. The distribution of adverse events by age and weight range, and treatment arm is shown in Table 3.

**Table 3:** Adverse events by age, weight range, and treatment arm

	Treatment group n (%)		
	ASMQ + Pq	CQ + Pq	AL + Pq
Age (years)			
> 59	14 (30.4)	21 (45.7)	11 (23.9)
18–39	190 (25.4)	255 (34.0)	304 (40.6)
39–59	199 (24.9)	367 (46.0)	232 (29.1)
Weight (kg)			
50–70	131 (20.2)	238 (36.8)	278 (43.0)
70–80	118 (25.8)	214 (46.7)	126 (27.5)
80–90	154 (31.6)	191 (39.1)	143 (29.3)

749 events (41.0%) were reported as possible, probable/likely or highly probably related to the treatment drug; 48.9% were grade I, 35.1% were grade II, none grade III and one SAE (grade IV). The distribution of all adverse events according with their intensity and causality are presented at Table 4. The AE distribution per causality and treatment group is shown in Additional file 4.

**Table 4:** Distribution of all adverse events (1593) by intensity and causality

Grade	Causality n (%)					Total
	Doubtful	Unlikely	Possible	Probable/likely	Highly probable	
I	597 (37.5)	107 (6.7)	*538 (33.8)*	*118 (7.4)*	*19 (1.2)*	1379 (86.6)
II	86 (5.4)	49 (3.1)	*62 (3.9)*	*10 (0.6)*	*1 (0.1)*	208 (13.1)
IV	0 (0)	0 (0)	*0 (0)*	*1 (0.1)*	*0 (0)*	1 (0.1)
NA	3 (0.19)	2 (0.13)	*0 (0)*	*0 (0)*	*0 (0)*	5 (0.31)
Total	686 (43.1)	158 (9.9)	*600 (37.7)*	*129 (8.1)*	*20 (1.3)*	1593 (100)

Italic values indicate the relevant safety outcomes

The distribution of all adverse events according with their intensity and causality per treatment group is provided (see Additional file 5).

All adverse events (1593) were grouped based on the main body system affected and treatment allocation (see Additional file 6). Non-specific complaints were most common (483), followed by gastrointestinal (450). As these symptoms could reflect the clinical illness or an AE, they were only considered as AE, if they were not present before dosing or they got worse after treatment. This overlap with symptoms may have led to an over estimation of AE. Tables listing all AE with a frequency higher than 3% per regimen allocation are provided (see Additional file 7).

The study treatment AE profile was very similar between study groups with the only category showing any difference being the occurrence of insomnia and abdominal pain (p < 0.04 and p < 0.03 respectively), using Pearson’s Chi squared test.

**Fig. 4:**
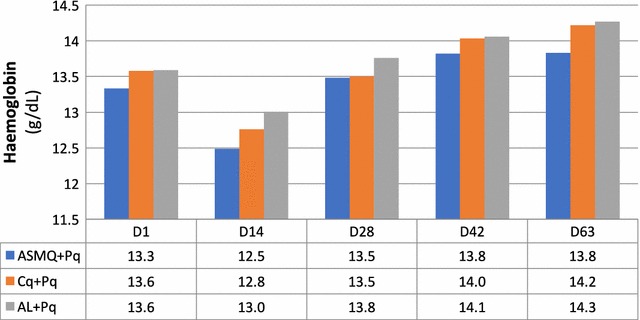
Haemoglobin per regimen allocation at day 0, 14, 28, 42, and 63

Changes in haemoglobin (Hb) were a secondary outcome. There was a slight decrease in all groups at day 14 followed by recovery (Fig. 4). The median difference in haemoglobin at day 14 (Hb at day 14 − Hb at baseline/Hb at baseline) [22] in ASMQ + Pq, AL + Pq, and CQ + Pq were − 0.74 (95% CI − 1.13; − 0.36), − 0.49 (95% CI − 0.87; − 0.09), and − 0.58 (95% CI − 0.91; − 0.24), respectively. These were not statistically different. A drop of more than 20% from baseline was observed in 6 patients in each of the arms, ASMQ + Pq and AL + Pq and in 3 CQ + Pq patients. No patient had a drop of more than 30% from baseline. The mean and range haemoglobin at day 0, 14, 28, 42, and 63 is shown in Additional file 8. Unfortunately, haemoglobin was not measured between 48 h and day 14; the lack of a day 7 measurement is a limitation of this study.

The effect of the study population’s baseline characteristics and the use of concomitant medication on the frequency of AE in each treatment arm was assessed. In this generalized linear model (Poisson distribution), the dependent variable was the count of possible, probable/likely or highly probable AE related to the study treatments per person. Adverse events were less common in males (OR 0.78; 95% CI 0.60–0.99; p = 0.04), but use of antipyretic or antihypertensive was associated with high rates of adverse events (OR 1.64; 95% CI 1.29–2.10; p < 0.01 and OR 1.55; 95% CI 0.92–2.44; p = 0.07, respectively).

### Exploratory analyses

This study was not powered to detect differences in cure rates between treatment regimens and no statistically significant difference could be detected between the treatment arms at any time point.

A generalized linear model (binominal distribution) was used to explore the influence of baseline variables and concomitant treatments upon failure at day 63, using a significance level of 5%. There was a strong association between the use of antipyretic medications and treatment failure (OR 3.2; 95% CI 1.3–8.3; p = 0.01). A trend towards an association between the use of anti-ulcerative drugs and treatment success was also observed (OR 0.12; 95% CI 0.001–0.73; p = 0.06).

The effect of the treatment on the time to clearance of gametocytes was evaluated in the three arms. Patients in the ASMQ + Pq and AL + Pq arm had all cleared gametocytes by day 2. In the CQ + Pq arm, 5 out of 43 patients (11.6%) had gametocytes present of day 2. Gametocytes only reappeared in five of the 103 patients, making meaningful conclusions impossible.

## Discussion

There has been recent international agreement that the importance of vivax malaria has been neglected [11, 23, 24], and that there is a need for new treatment approaches [25]. For most of the world, the first-line treatment recommendation of CQ + Pq has not changed since the 50s [11], despite the concerns about the emergence of CQ-resistance [17]. Standard treatment has been based upon the need to clear both the red cell forms and the hepatic forms and the relatively slow emergence of resistance of *P. vivax* to chloroquine may be due to the fact that standard CQ + Pq treatment has been an effective combination therapy [1]. In addition to clearing hypnozoites, primaquine is active against blood stages of vivax [26] and chloroquine use also increases primaquine blood levels [27]; concomitant use of chloroquine and primaquine is more effective than sequential use [28].

ACT has been recommended by the WHO to treat vivax since 2010 [29], as it appears to have equivalent *P. vivax* schizonticidal activity to chloroquine [30] and it is recommended as the first-line treatment in areas of CQ-resistant *P. vivax* emerges. There are compelling arguments to look for a simpler and unified ACT treatment for both species of malaria. A single radical treatment would be useful in co-endemic areas where species diagnosis is difficult [31] or mixed infections occur that are misdiagnosed as *P. falciparum*. The use of ACT and primaquine for the treatment of *P. falciparum* infection also has the advantage of eradicating hypnozoites and preventing relapses from previous *P. vivax* infection that can happen following a *P. falciparum* infection [32]. There are also considerable logistic advantages for the use of ACT to treat both falciparum and vivax malaria with efficiencies of stock and supply management and an incentive to maintain ACT production as the requirement for falciparum treatment courses reduces.

In this study, all three treatment arms demonstrated cure rates > 90% in all treatment arms at day 42 but by day 63, cure rates had dropped below 90% (although all the 95% CI includes the 90% cure rate). It demonstrates the importance of a longer follow-up time in detecting failures, particularly in vivax when reinfection or relapse can occur [33]. This study did not detect any advantage from the use of an ACT with a longer half-life partner drugs, as has been shown previously; this may be due to the concomitant use of primaquine in our study [34]. Further studies with PCR analysis in an attempt to discriminate reinfection from relapse would be of interest.

Drug interactions and the safety profile are important factors in choosing the optimum first line treatment, in addition to pill burden and food restrictions. This trial evaluated the efficacy and safety of three vivax treatment regimens, and provides additional reassurance about the safety of ASMQ and AL when given with primaquine; information that is considered important by WHO as strategies for control and elimination of *P. vivax* malaria are developed [1]. Most of the AE with ACT regimens were mild to moderate (CTCAE grade I or II), and required minimal intervention. The frequencies of possibly, probably/likely or highly probably related AE did not substantially vary between the CQ + Pq regimen and the ACT + Pq regimens.

Despite this low level of adverse events, the ACT and primaquine combination still has significant drawbacks. Its use is limited in pregnancy, breastfeeding and G6DP-deficient population. The one SAE in this study was related to G6PD deficiency. These restrictions impose serious limitations to its use as an elimination tool in many parts of the world, although the primaquine regimen used in this study is the 7-day regimen currently recommended by the Brazilian National Malaria Control Programme. This regimen delivers a total dose from 3 to 4.2 mg/kg [13]. This is the WHO recommended total dose (although suggested over 14 days) and this regimen retains efficacy in some parts of the world [35–37]. The 7 day regimen has been suggested to improve adherence [13], although this has never been clearly demonstrated.

This study was not sufficiently powered to detect differences between the three arms and only followed up patients for 63 days, meaning that late relapses would not have been detected. The study population did not include children, limiting the conclusions of efficacy in this populations. The absence of PCR also meant that relapses could not be distinguished from reinfection; a critical issue in assessing vivax treatment. Nevertheless, this study demonstrates the efficacy and safety of two ACT and CQ in combination with 7 days of primaquine to treat uncomplicated vivax malaria in Brazil and demonstrated the feasibility and utility of a standardized treatment approach for all malaria cases.

## Additional files

**Additional file 1: Table S1.** Reasons to not be included—Consort Diagram.

**Additional file 2: Table S2.** Distribution of use of concomitant medication (grouped in therapeutic class) in each study arm, parenthesis presents the percentage of the line. **Figure S1.** Distribution of most frequent medications used per study visit and treatment group.

**Additional file 3: Table S3.** Proportion of treatment success per treatment arm in ITT population (n = 88 per arm) at day 28, 42 and 63. **Table S4.** Proportion of treatment success per treatment arm in PP population at day 07, 14 and 21. **Table S5.** Proportion of treatment success per treatment arm in ITT population (n = 88 per arm) at day 07, 14 and 21.

**Additional file 4: Table S6.** Distribution of adverse events per causality and treatment group.

**Additional file 5: Table S7.** Distribution of adverse events per causality and intensity (grade) in the treatment group ASMQ + Pq. **Table S8.** Distribution of adverse events per causality and intensity (grade) in the treatment group CQ + Pq. **Table S9.** Distribution of adverse events per causality and intensity (grade) in the treatment group AL + Pq.

**Additional file 6: Table S10.** All adverse events (1593) per body system and treatment allocation.

**Additional file 7: Table S11.** Adverse events with frequency higher than 3% in the ASMQ + Pq arm (n total = 403). **Table S12.** Adverse events with frequency higher than 3% in the CQ + Pq arm (n total = 643). **Table S13.** Adverse events with frequency higher than 3% in the AL + Pq arm (n total = 547).

**Additional file 8: Table S14.** Haemoglobin mean per regimen allocation at day 0, 14, 28, 42, and 63.

## Abbreviations

ACT: artemisinin-based combination therapy
AE: adverse events
AL: fixed dose combination of 20 mg + 120 mg artemether and lumefantrine
AL + Pq: fixed dose combination artemether and lumefantrine with concomitant use of primaquine
ASMQ: fixed dose combination of 100 mg + 200 mg artesunate and mefloquine
ASMQ + Pq: fixed dose combination artesunate and mefloquine with concomitant use of primaquine
CEPEM: Tropical Medicine Research Centre of Rondônia
CI: confidence interval
CNS: Brazilian National Health Council
CONEP: Brazilian National Council on Ethics in Research
CQ: chloroquine
CQ + Pq: chloroquine with concomitant use of primaquine
CTCAE: Common Terminology Criteria for Adverse Events
FIOCRUZ: Oswaldo Cruz Foundation
FMT: Fundação de Medicina Tropical Doutor Heitor Vieira
G6PD: glucose-6-phosphate dehydrogenase
GCP: good clinical practice
hCG: human chorionic gonadotropin
ITT: intention-to-treat
PCR: polymerase chain reaction
PP: per protocol
SD: standard deviations
SEFAR: Laboratory of Pharmacokinetics
SISNEP: National System of Ethics in Research
UNIR: Federal University of Rondônia
WHO: World Health Organization

## References

[1] WHO (2015). Control and elimination of vivax malaria: a technical brief.

[2] Gething PW, Elyazar IR, Moyes CL, Smith DL, Battle KE, Guerra CA (2012). A long neglected world malaria map: *Plasmodium vivax* endemicity in 2010. PLoS Negl Trop Dis.

[3] Lima ID, Lapouble OM, Duarte EC (2017). Time trends and changes in the distribution of malaria cases in the Brazilian Amazon Region, 2004–2013. Mem Inst Oswaldo Cruz.

[4] Ferreira MU, Castro MC (2016). Challenges for malaria elimination in Brazil. Malar J.

[5] Siqueira AM, Mesones-Lapouble O, Marchesini P, de Souza Sampaio V, Brasil P, Tauil PL (2016). *Plasmodium vivax* landscape in Brazil: scenario and challenges. Am J Trop Med Hyg.

[6] Secretaria de Vigilância em Saúde. Malária: Monitoramento dos casos no Brasil em 2014. Boletim Epidemiológico Secretaria de Vigilância em Saúde Ministério da Saúde; 2015. p. 46.

[7] Rahimi BA, Thakkinstian A, White NJ, Sirivichayakul C, Dondorp AM, Chokejindachai W (2014). Severe vivax malaria: a systematic review and meta-analysis of clinical studies since 1900. Malar J.

[8] Kochar DK, Das A, Kochar SK, Saxena V, Sirohi P, Garg S (2009). Severe *Plasmodium vivax* malaria: a report on serial cases from Bikaner in northwestern India. Am J Trop Med Hyg.

[9] Naing C, Whittaker MA, Nyunt Wai V, Mak JW (2014). Is *Plasmodium vivax* malaria a severe malaria?: a systematic review and meta-analysis. PLoS Negl Trop Dis.

[10] Vitor-Silva S, Reyes-Lecca RC, Pinheiro TR, Lacerda MV (2009). Malaria is associated with poor school performance in an endemic area of the Brazilian Amazon. Malar J.

[11] Mendis K, Sina BJ, Marchesini P, Carter R (2001). The neglected burden of *Plasmodium vivax* malaria. Am J Trop Med Hyg.

[12] Botto-Menezes C, Bardaji A, Dos Santos Campos G, Fernandes S, Hanson K, Martinez-Espinosa FE (2016). Costs associated with malaria in pregnancy in the Brazilian Amazon, a low endemic area where *Plasmodium vivax* predominates. PLoS Negl Trop Dis.

[13] Ministério da Saúde do Brasil. Guia prático de tratamento de malária. In: Série A Normas e Manuais Técnicos. Secretária de Vigilância em Saúde—Ministério da Saúde do Brasil; 2010. http://www.saude.gov.br/bvs. Accessed June 2017.

[14] Gomes Mdo S, Vieira JL, Machado RL, Nacher M, Stefani A, Musset L (2015). Efficacy in the treatment of malaria by *Plasmodium vivax* in Oiapoque, Brazil, on the border with French Guiana: the importance of control over external factors. Malar J.

[15] Pereira D, Daher A, Zanini G, Maia I, Fonseca L, Pitta L (2016). Safety, efficacy and pharmacokinetic evaluations of a new coated chloroquine tablet in a single-arm open-label non-comparative trial in Brazil: a step towards a user-friendly malaria vivax treatment. Malar J.

[16] Negreiros S, Farias S, Viana GMR, Okoth SA, Chenet SM, De Souza TMH (2016). Efficacy of chloroquine and primaquine for the treatment of uncomplicated *Plasmodium vivax* malaria in Cruzeiro do Sul, Brazil. Am J Trop Med Hyg.

[17] Price RN, von Seidlein L, Valecha N, Nosten F, Baird JK, White NJ (2014). Global extent of chloroquine-resistant *Plasmodium vivax*: a systematic review and meta-analysis. Lancet Infect Dis.

[18] WHO (2009). Methods for surveillance of antimalarial drug efficacy.

[19] ICH. Good clinical practice. International conference on harmonisation of technical requirements for registration of pharmaceuticals for human use. 1996. http://www.ich.org/fileadmin/Public_Web_Site/ICH_Products/Guidelines/Efficacy/E6/E6_R1_Guideline.pdf. Accessed June 2017.

[20] Pan American Health Organization. Boas Práticas Clínicas: Documento das Américas. Washington, DC: Pan American Health Organization WHO; 2005. http://www.anvisa.gov.br/medicamentos/pesquisa/boaspraticas_americas.pdf. Accessed June 2017.

[21] NCI common terminology criteria for adverse events. http://ctep.cancer.gov/protocolDevelopment/electronic_applications/ctc.htm-ctc_40. Accessed June 2017.

[22] Uthman OA, Graves PM, Saunders R, Gelband H, Richardson M, Garner P (2017). Safety of primaquine given to people with G6PD deficiency: systematic review of prospective studies. Malar J.

[23] Carlton JM, Sina BJ, Adams JH (2011). Why is *Plasmodium vivax* a neglected tropical disease?. PLoS Negl Trop Dis.

[24] Baird JK (2007). Neglect of *Plasmodium vivax* malaria. Trends Parasitol.

[25] Price RN, Douglas NM, Anstey NM, von Seidlein L (2011). *Plasmodium vivax* treatments: what are we looking for?. Curr Opin Infect Dis.

[26] Pukrittayakamee S, Vanijanonta S, Chantra A, Clemens R, White NJ (1994). Blood stage antimalarial efficacy of primaquine in *Plasmodium vivax* malaria. J Infect Dis.

[27] Pukrittayakamee S, Tarning J, Jittamala P, Charunwatthana P, Lawpoolsri S, Lee SJ (2014). Pharmacokinetic interactions between primaquine and chloroquine. Antimicrob Agents Chemother.

[28] Baird JK, Hoffman SL (2004). Primaquine therapy for malaria. Clin Infect Dis.

[29] WHO (2010). Guidelines for the treatment of malaria.

[30] Gogtay N, Kannan S, Thatte UM, Olliaro PL, Sinclair D. Artemisinin-based combination therapy for treating uncomplicated *Plasmodium vivax* malaria. Cochrane Database Syst Rev. 2013;(10):CD008492. 10.1002/14651858.CD008492.pub3. http://onlinelibrary.wiley.com/doi/10.1002/14651858.CD008492.pub3/abstract;jsessionid=49E497269900A8965B3F62DEEB6D81A5.f02t02. Accessed 18 Jan 2018. 10.1002/14651858.CD008492.pub3 PMC6532731 24163021

[31] Douglas NM, Anstey NM, Angus BJ, Nosten F, Price RN (2010). Artemisinin combination therapy for vivax malaria. Lancet Infect Dis.

[32] White NJ (2011). Determinants of relapse periodicity in *Plasmodium vivax* malaria. Malar J.

[33] Stepniewska K, Taylor WR, Mayxay M, Price R, Smithuis F, Guthmann JP (2004). In vivo assessment of drug efficacy against *Plasmodium falciparum* malaria: duration of follow-up. Antimicrob Agents Chemother.

[34] Visser BJ, Wieten RW, Kroon D, Nagel IM, Belard S, van Vugt M (2014). Efficacy and safety of artemisinin combination therapy (ACT) for non-falciparum malaria: a systematic review. Malar J.

[35] Durand S, Cabezas C, Lescano AG, Galvez M, Gutierrez S, Arrospide N (2014). Efficacy of three different regimens of primaquine for the prevention of relapses of *Plasmodium vivax* malaria in the Amazon Basin of Peru. Am J Trop Med Hyg.

[36] Abdon NP, Pinto AY, das Silva Rdo S, de Souza JM (2001). Assessment of the response to reduced treatment schemes for vivax malaria. Rev Soc Bras Med Trop.

[37] John GK, Douglas NM, von Seidlein L, Nosten F, Baird JK, White NJ (2012). Primaquine radical cure of *Plasmodium vivax*: a critical review of the literature. Malar J.

