# Supplementary material for: Efficacy and safety of artemisinin-based combination therapy and chloroquine with concomitant primaquine to treat Plasmodium vivax malaria in Brazil: an open label randomized clinical trial
Source: Malar J. 2018 Jan 24;17:45. doi: 10.1186/s12936-018-2192-x (PMC5782374; doi:10.1186/s12936-018-2192-x)
Supplement: Supplementary file 1 — Additional file 1: Table S1. Reasons to not be included—Consort Diagram. [file 12936_2018_2192_MOESM1_ESM.docx]

| **Reasons to not be included** | **numbers of patients (%)** | |
| --- | --- | --- |
| Unavailability for follow-up | 492 | (19.9%) |
| Parasitaemia lower than 250/μL | 394 | (15.9%) |
| Malaria treatment within the past 63 days | 376 | (15.2%) |
| Residency in rural area | 187 | (7.6%) |
| Weight range, i.e., <50kg or > 90kg | 139 | (5.6%) |
| Age range, i.e., < 18 or > 70 years old | 122 | (4.9%) |
| Falciparum malaria or mixed infection | 113 | (4.6%) |
| Other | 89 | (3.6%) |
| Declined to participate | 69 | (2.8%) |
| Pregnancy | 44 | (1.8%) |
| Haemoglobin < 7 g/dL | 03 | (0.1%) |

**Table S1** Reasons to not be included- Consort Diagram
