# Supplementary material for: Efficacy and safety of artemisinin-based combination therapy and chloroquine with concomitant primaquine to treat Plasmodium vivax malaria in Brazil: an open label randomized clinical trial
Source: Malar J. 2018 Jan 24;17:45. doi: 10.1186/s12936-018-2192-x (PMC5782374; doi:10.1186/s12936-018-2192-x)
Supplement: Supplementary file 2 — Additional file 2: Table S2. Distribution of use of concomitant medication (grouped in therapeutic class) in each study arm, parenthesis presents the percentage of the line. Figure S1. Distribution of most frequent medications used per study visit and treatment group. [file 12936_2018_2192_MOESM2_ESM.docx]

**Table S2. Distribution of use of concomitant medication (grouped in therapeutic class) in each study arm, parenthesis presents the percentage of the line**

| **Therapeutic class**  **of concomitant**  **medication** | | | **study arm**  **N (%)** | | | | | | |  |
| --- | --- | --- | --- | --- | --- | --- | --- | --- | --- | --- |
|  |  |  |  | | **ASMQ+Pq** | **Cq+Pq** | | **AL+Pq** | |  |
| **Total** |  |  |  | | 115(23.23) | 189(38.18) | | 191(38.59) | |  |
|  |  |  |  | |  |  | |  | |  |
|  |  |  |  | |  |  | |  | |  |
|  |  | analgesic/ antipyretic | |  | 74(25.78) | | 117(40.77) | | 96(33.45) | |
|  |  | antispasmodic | |  | 5(17.24) | | 3(10.34) | | 21(72.41) | |
|  |  | antifungal | |  | 1(33.33) | | 1(33.33) | | 1(33.33) | |
|  |  | antiemetic | |  | 7(29.17) | | 3(12.5) | | 14(58.33) | |
|  |  | antihistaminic | |  | 2(33.33) | | 4(66.67) | | 0(0) | |
|  |  | antipsychotic | |  | 0(0) | | 1(100) | | 0(0) | |
|  |  | antiulcer | |  | 5(14.29) | | 10(28.57) | | 20(57.14) | |
|  |  | antibiotic | |  | 4(19.05) | | 7(33.33) | | 10(47.62) | |
|  |  | antidepressant | |  | 0(0) | | 1(50) | | 1(50) | |
|  |  | antihypertensive | |  | 4(10.53) | | 21(55.26) | | 13(34.21) | |
|  |  | anti-vertigo | |  | 0(0) | | 1(50) | | 1(50) | |
|  |  | antiviral (topic) | |  | 1(50) | | 0(0) | | 1(50) | |
|  |  | anti-dyslipidaemia | |  | 1(50) | | 1(50) | | 0(0) | |
|  |  | expectorant | |  | 0(0) | | 5(100) | | 0(0) | |
|  |  | hepatic-protector | |  | 4(36.36) | | 3(27.27) | | 4(36.36) | |
|  |  | hydration | |  | 1(100) | | 0(0) | | 0(0) | |
|  |  | hypoglycaemic drug | |  | 0(0) | | 6(54.55) | | 5(45.45) | |
|  |  | traditional drugs | |  | 2(40) | | 1(20) | | 2(40) | |
|  |  | vitamins | |  | 4(40) | | 4(40) | | 2(20) | |

**Figure S1.** Distribution of most frequent medications used per study visit and treatment group

| 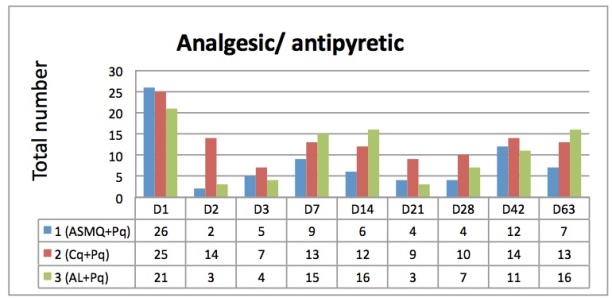 | 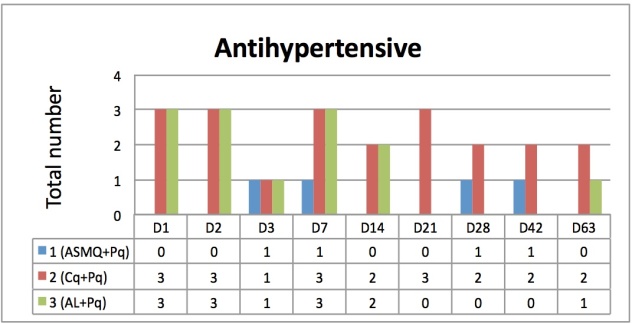 |
| --- | --- |
| 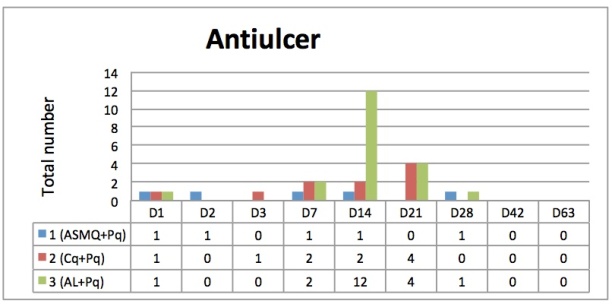 | 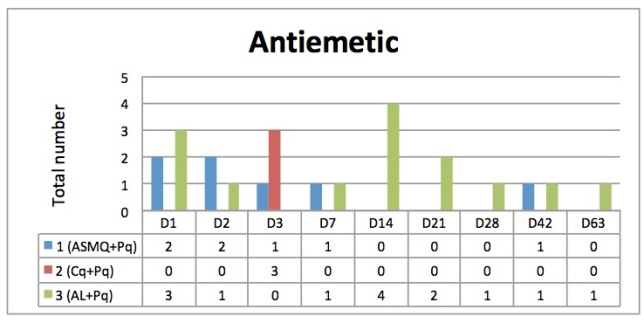 |
