# Supplementary material for: Efficacy and safety of artemisinin-based combination therapy and chloroquine with concomitant primaquine to treat Plasmodium vivax malaria in Brazil: an open label randomized clinical trial
Source: Malar J. 2018 Jan 24;17:45. doi: 10.1186/s12936-018-2192-x (PMC5782374; doi:10.1186/s12936-018-2192-x)
Supplement: Supplementary file 3 — Additional file 3: Table S3. Proportion of treatment success per treatment arm in ITT population (n = 88 per arm) at day 28, 42 and 63. Table S4. Proportion of treatment success per treatment arm in PP population at day 07, 14 and 21. Table S5. Proportion of treatment success per treatment arm in ITT population (n = 88 per arm) at day 07, 14 and 21. [file 12936_2018_2192_MOESM3_ESM.docx]

**Table S3.** Proportion of treatment success per treatment arm in ITT population (n=88 per arm) at day 28, 42 and 63.

| **Visit day** | **Study treatment** | | | | | |
| --- | --- | --- | --- | --- | --- | --- |
|  | **ASMQ+Pq** | | **CQ+Pq** | |  | **AL+Pq** |
|  | **%** | **IC 95%** | **%** | **IC 95%** | **%** | **IC 95%** |
|  |  |  |  |  |  |  |
| **D28** | 97% | [91-100] | 93% | [88-98] | 92% | [86-98] |
|  |  |  |  |  |  |  |
| **D42** | 92% | [86-98] | 86% | [79-94] | 88% | [81-94] |
|  |  |  |  |  |  |  |
| **D63** | 76% | [67-85] | 81% | [72-89] | 77% | [69-86] |

**Table S4.** Proportion of treatment success per treatment arm in PP population at day 07, 14 and 21.

| **Visit day** | **Study treatment** | | | | | |
| --- | --- | --- | --- | --- | --- | --- |
|  | **ASMQ+Pq** | | **Cq+Pq** | | **AL+Pq** | |
|  | **%**  **(n)** | **IC 95%** | **%**  **(n)** | **IC 95%** | **%**  **(n)** | **IC 95%** |
|  |  |  |  |  |  |  |
| **D07** | 100%  (87) | - | 100%  (85) | - | 100%  (86) | - |
| **D14** |  |  |  |  |  |  |
|  | 100%  (87) | - | 100%  (84) | - | 100%  (84) | - |
| **D21** |  |  |  |  |  |  |
|  | 100%  (86) | - | 100%  (84) | - | 99%  (84) | [97-101] |

**Table S5.** Proportion of treatment success per treatment arm in ITT population (n=88 per arm) at day 07, 14 and 21.

| **Visit day** | **Study treatment** | | | | | |
| --- | --- | --- | --- | --- | --- | --- |
|  | **ASMQ+Pq** | | **CQ+Pq** | |  | **AL+Pq** |
|  | **%** | **IC 95%** | **%** | **IC 95%** | **%** | **IC 95%** |
|  |  |  |  |  |  |  |
| **D07** | 99% | [97-101] | 98% | [95-101] | 98% | [95-101] |
|  |  |  |  |  |  |  |
| **D14** | 99% | [97-101] | 97% | [93-101] | 98% | [95-101] |
|  |  |  |  |  |  |  |
| **D21** | 98% | [95-101] | 95% | [91-100] | 94% | [89-99] |
