# Supplementary material for: Efficacy and safety of artemisinin-based combination therapy and chloroquine with concomitant primaquine to treat Plasmodium vivax malaria in Brazil: an open label randomized clinical trial
Source: Malar J. 2018 Jan 24;17:45. doi: 10.1186/s12936-018-2192-x (PMC5782374; doi:10.1186/s12936-018-2192-x)
Supplement: Supplementary file 4 — Additional file 4: Table S6. Distribution of adverse events per causality and treatment group. [file 12936_2018_2192_MOESM4_ESM.docx]

**Table S6** Distribution of adverse events per causality and treatment group.

| **Causality** | |  |  | **Treatment group**  **n (%)** | | | |
| --- | --- | --- | --- | --- | --- | --- | --- |
|  |  |  |  | **ASMQ+Pq** | **CQ+Pq** | **AL+Pq** | **Total** |
|  |  | **Doubtful** |  | 160 (23.3) | 283 (41.2) | 243 (35.4) | 686 |
|  |  | **Unlikely** |  | 35 (22.1) | 70 (44.3) | 53 (33.5) | 158 |
|  |  | **Possible** |  | 163 (27.2) | 238 (39.7) | 199 (33.2) | 600 |
|  |  | **Probable/ Likely** |  | 36 (27.9) | 43 (33.3) | 50 (38.8) | 129 |
|  |  | **Highly Probable** |  | 9 (45) | 9 (45) | 2 (10) | 20 |
