# Supplementary material for: Efficacy and safety of artemisinin-based combination therapy and chloroquine with concomitant primaquine to treat Plasmodium vivax malaria in Brazil: an open label randomized clinical trial
Source: Malar J. 2018 Jan 24;17:45. doi: 10.1186/s12936-018-2192-x (PMC5782374; doi:10.1186/s12936-018-2192-x)
Supplement: Supplementary file 5 — Additional file 5: Table S7. Distribution of adverse events per causality and intensity (grade) in the treatment group ASMQ + Pq. Table S8. Distribution of adverse events per causality and intensity (grade) in the treatment group CQ + Pq. Table S9. Distribution of adverse events per causality and intensity (grade) in the treatment group AL + Pq. [file 12936_2018_2192_MOESM5_ESM.docx]

**Table S7.** Distribution of adverse events per causality and intensity (grade) in the treatment group ASMQ +Pq

| **Grade** | **Causality (N,%)**  **ASMQ+Pq** | | | | |  |
| --- | --- | --- | --- | --- | --- | --- |
|  | **Doubtful** | **Unlikely** | **Possible** | **Probable/ Likely** | **Highly Probable** | **Total** |
| **1** | 141(34.99) | 27(6.7) | 151(37.47) | 34(8.44) | 8(1.99) | 361(89.58) |
| **2** | 18(4.47) | 8(1.99) | 12(2.98) | 2(0.5) | 1(0.25) | 41(10.17) |
| **4** | 0(0) | 0(0) | 0(0) | 0(0) | 0(0) | 0(0) |
| **NC*** | 1(0.25) | 0(0) | 0(0) | 0(0) | 0(0) | 1(0.25) |
| **Total** | 160(39.7) | 35(8.68) | 163(40.45) | 36(8.93) | 9(2.23) | 403(100) |

* Non-classified

**Table S8.** Distribution of adverse events per causality and intensity (grade) in the treatment group CQ+Pq

| **Grade** | **Causality (N,%)**  **CQ+Pq** | | | | |  |
| --- | --- | --- | --- | --- | --- | --- |
|  | **Doubtful** | **Unlikely** | **Possible** | **Probable/ Likely** | **Highly Probable** | **Total** |
| **1** | 254  (39.5) | 52  (8.09) | 212 (32.97) | 37  (5.75) | 9  (1.4) | 564  (87.71) |
| **2** | 28(4.35) | 17(2.64) | 26(4.04) | 5(0.78) | 0(0) | 76  (11.82) |
| **4** | 0(0) | 0(0) | 0(0) | 1(0.16) | 0(0) | 1(0.16) |
| **NC*** | 1(0.16) | 1(0.16) | 0(0) | 0(0) | 0(0) | 2(0.31) |
| **Total** | 283  (44.01) | 70  (10.89) | 238 (37.01) | 43  (6.69) | 9  (1.4) | 643  (100) |

* Non-classified

**Table S9.** Distribution of adverse events per causality and intensity (grade) in the treatment group AL+Pq.

| **Grade** | **Causality (N,%)**  **AL+Pq** | | | | |  |
| --- | --- | --- | --- | --- | --- | --- |
|  | **Doubtful** | **Unlikely** | **Possible** | **Probable/ Likely** | **Highly Probable** | **Total** |
| **1** | 202(36.93) | 28(5.12) | 175 (31.99) | 47(8.59) | 2(0.37) | 454(83) |
| **2** | 40(7.31) | 24(4.39) | 24(4.39) | 3(0.55) | 0(0) | 91 (16.64) |
| **4** | 1(0.18) | 1(0.18) | 0(0) | 0(0) | 0(0) | 2(0.37) |
| **Total** | 243(44.42) | 53(9.69) | 199 (36.38) | 50(9.14) | 2(0.37) | 547(100) |
