# Supplementary material for: Efficacy and safety of artemisinin-based combination therapy and chloroquine with concomitant primaquine to treat Plasmodium vivax malaria in Brazil: an open label randomized clinical trial
Source: Malar J. 2018 Jan 24;17:45. doi: 10.1186/s12936-018-2192-x (PMC5782374; doi:10.1186/s12936-018-2192-x)
Supplement: Supplementary file 6 — Additional file 6: Table S10. All adverse events (1593) per body system and treatment allocation. [file 12936_2018_2192_MOESM6_ESM.docx]

**Table S10.** All adverse events (1,593) per body system and treatment allocation

|  |  |  |  | **Treatment allocation**  **n (%)** | | |  |
| --- | --- | --- | --- | --- | --- | --- | --- |
| **Variable** | | |  | **ASMQ+Pq** | **CQ+Pq** | **AL+Pq** | **Total** |
| **Total** |  |  |  | 403(25.3) | 643(40.36) | 547(34.34) | 1593 |
|  |  |  |  |  |  |  |  |
| **Body System** |  |  |  |  |  |  |  |
|  |  | Cardiovascular |  | 4(28.57) | 3(21.43) | 7(50) | 14 |
|  |  | Dermatological |  | 28(25) | 55(49.11) | 29(25.89) | 112 |
|  |  | Digestive |  | 110(24.44) | 158(35.11) | 182(40.44) | 450 |
|  |  | General Status |  | 115(23.81) | 203(42.03) | 165(34.16) | 483 |
|  |  | Excretory |  | 1(10) | 7(70) | 2(20) | 10 |
|  |  | Musculoskeletal |  | 28(25) | 43(38.39) | 41(36.61) | 112 |
|  |  | Central nervous System |  | 77(31.3) | 107(43.5) | 62(25.2) | 246 |
|  |  | Peripheral nervous System |  | 3(50) | 1(16.67) | 2(33.33) | 6 |
|  |  | Reproductive female |  | 2(66.67) | 0(0) | 1(33.33) | 3 |
|  |  | Respiratory |  | 34(22.08) | 66(42.86) | 54(35.06) | 154 |
|  |  | Trauma |  | 1(33.33) | 0(0) | 2(66.67) | 3 |
