# Supplementary material for: Efficacy and safety of artemisinin-based combination therapy and chloroquine with concomitant primaquine to treat Plasmodium vivax malaria in Brazil: an open label randomized clinical trial
Source: Malar J. 2018 Jan 24;17:45. doi: 10.1186/s12936-018-2192-x (PMC5782374; doi:10.1186/s12936-018-2192-x)
Supplement: Supplementary file 7 — Additional file 7: Table S11. Adverse events with frequency higher than 3% in the ASMQ + Pq arm (n total = 403). Table S12. Adverse events with frequency higher than 3% in the CQ + Pq arm (n total = 643). Table S13. Adverse events with frequency higher than 3% in the AL + Pq arm (n total = 547). [file 12936_2018_2192_MOESM7_ESM.docx]

**Table S11.** Adverse events with frequency higher than 3% in the ASMQ+Pq arm (n total=403)

| **ASMQ+Pq** | |
| --- | --- |
| **Symptoms** | **n(%)** |
| headache | 51(12.66) |
| insomnia | 39(9.68) |
| nauseas | 36(8.93) |
| asthenia | 31(7.69) |
| dizziness | 28(6.95) |
| dyspnoea | 27(6.7) |
| pruritus | 24(5.96) |
| abdominal pain | 22(5.46) |
| muscle articular pain | 21(5.21) |
| vomitus | 16(3.97) |

**Table S12.** Adverse events with frequency higher than 3% in the **CQ+Pq** arm (n total=643)

| **Cq+Pq** | |
| --- | --- |
|  |  |
| **Symptoms** | **n(%)** |
| headache | 84(13.06) |
| insomnia | 62(9.64) |
| pruritus | 53(8.24) |
| abdominal pain | 52(8.09) |
| asthenia | 49(7.62) |
| nauseas | 42(6.53) |
| anorexia | 39(6.07) |
| dyspnoea | 38(5.91) |
| muscle articular pain | 32(4.98) |
| cough | 22(3.42) |

**Table S13.** Adverse events with frequency higher than 3% in the **AL+Pq** arm (n total=547)

| **AL+Pq** | |
| --- | --- |
|  |  |
| **Symptoms** | n**(%)** |
| headache | 62(11.33) |
| abdominal pain | 57(10.42) |
| asthenia | 54(9.87) |
| nauseas | 40(7.31) |
| dyspnoea | 33(6.03) |
| vomitus | 33(6.03) |
| insomnia | 32(5.85) |
| pruritus | 26(4.75) |
| muscle articular pain | 25(4.57) |
| epigastric pain | 24(4.39) |
| cough | 21(3.84) |
| anorexia | 19(3.47) |
| dizziness | 17(3.11) |
