# Supplementary material for: Efficacy and safety of artemisinin-based combination therapy and chloroquine with concomitant primaquine to treat Plasmodium vivax malaria in Brazil: an open label randomized clinical trial
Source: Malar J. 2018 Jan 24;17:45. doi: 10.1186/s12936-018-2192-x (PMC5782374; doi:10.1186/s12936-018-2192-x)
Supplement: Supplementary file 8 — Additional file 8: Table S14. Haemoglobin mean per regimen allocation at day 0, 14, 28, 42, and 63. [file 12936_2018_2192_MOESM8_ESM.docx]

**Table S14.** Haemoglobin mean per regimen allocation at day 0, 14, 28, 42, and 63

| **Days** | **Regimen allocation** | | | | | |
| --- | --- | --- | --- | --- | --- | --- |
|  | **ASMQ+Pq** | | **CQ+Pq** | | **AL+Pq** | |
|  | Mean  (SD) | Range  (min-max) | Mean  (SD) | Range  (min-max) | Mean (SD) | Range  (min-max)) |
| **D1** | 13.33  (1.66) | (8.6-17.3) | 13.58  (2.6) | (8.7-32.6) | 13.59  (2.01) | (9-18.2) |
| **D14** | 12.49  (1.46) | (9.9-16.2) | 12.76  (1.33) | (10.3-16.4) | 13.01  (1.42) | (8.4-16.3) |
| **D28** | 13.48  (1.27) | (10.9-16) | 13.5  (1.17) | (10.7-16.3) | 13.76 (1.2) | (10.1-16.2) |
| **D42** | 13.82  (1.18) | (11.5-16.4) | 14.03  (1.45) | (10.3-18.8) | 14.06 (1.3) | (10.4-17.1) |
| **D63** | 13.83  (1.33) | (11.1-16.4) | 14.22  (1.41) | (10.9-17.6) | 14.27 (1.15) | (11.3-16.7) |
